# Supplementary material for: Adolescent and Juvenile Idiopathic Scoliosis: Which Patients Obtain Good Results with 12 Hours of Cheneau–Toulouse–Munster Nighttime Bracing?
Source: Children (Basel). 2022 Jun 17;9(6):909. doi: 10.3390/children9060909 (PMC9221823; doi:10.3390/children9060909)
Supplement: Supplementary file 1 [file children-09-00909-s001.zip › children-1709541-Supplementary Material S1.pdf]

SUPPLEMENTAL FILE S1

<table>
<tr><th colspan="2">FRONTAL PLANE</th></tr>
<tr><td>Frontal C7 Tilt (mm and degree)</td><td>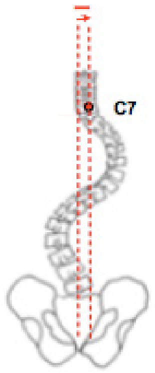

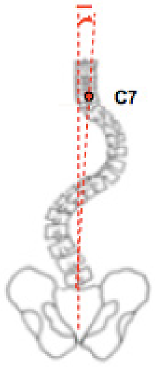
</td></tr>
<tr><td>Acromio-femoral Angle</td><td>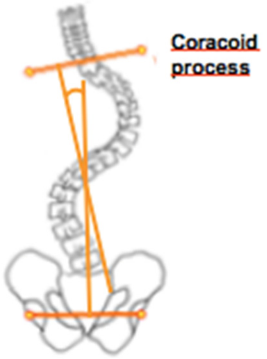
</td></tr>
</table>

Vertebrae Number	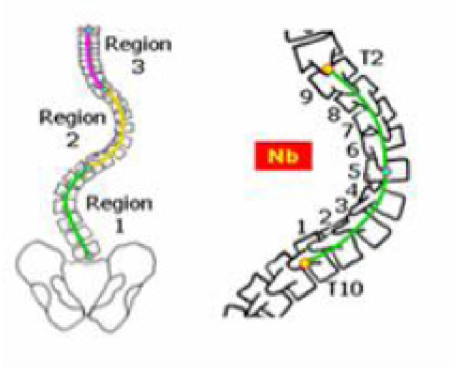
Lateral Displacement Apex Vertebrae	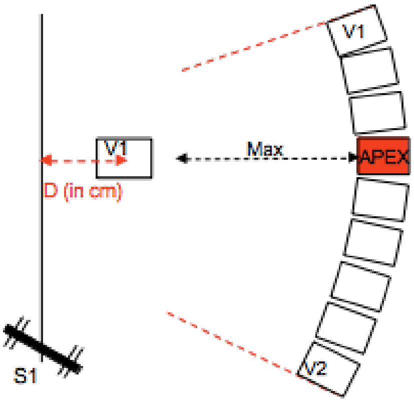
Iliolumbar Angle	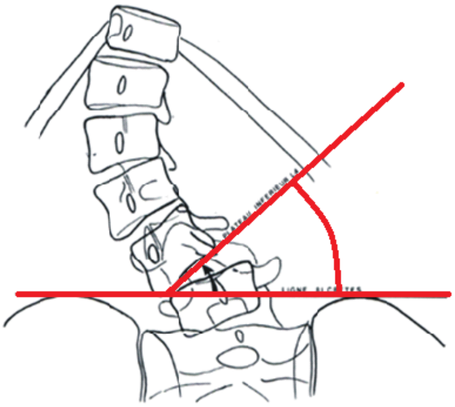
Wedging	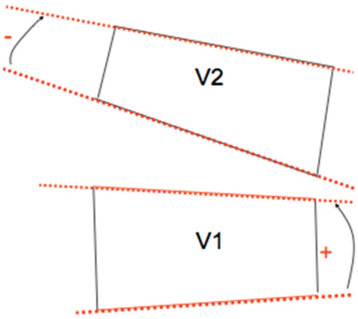

<table>
<tr><th colspan="2">SAGITTAL PLANE</th></tr>
<tr><td>Sagittal C7 Tilt degree</td><td>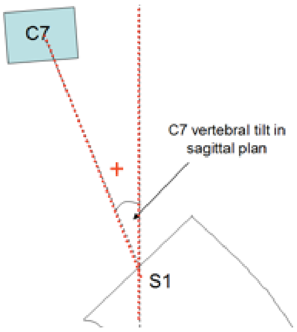
</td></tr>
<tr><td>Pelvic Incidence</td><td>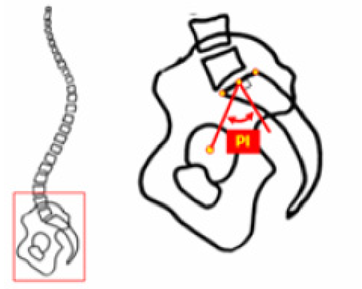
</td></tr>
<tr><td>Sacral Slope</td><td>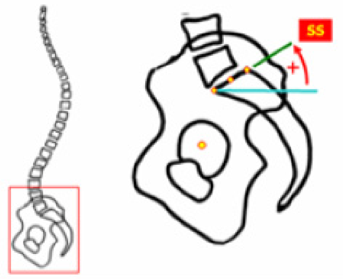
</td></tr>
<tr><td>Pelvic Tilt</td><td>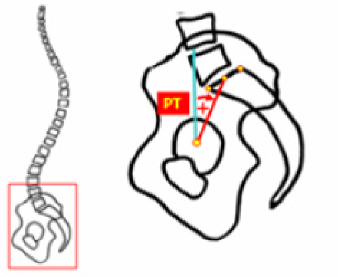
</td></tr>
<tr><td>Lumbar Lordosis and Thoracic Kyphosis</td><td>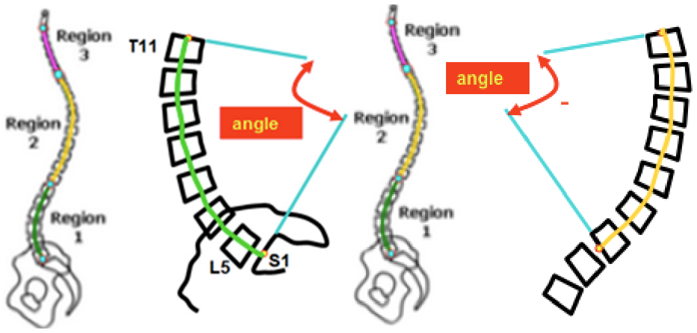
</td></tr>
</table>
